# Shared Effects of Genetic and Intrauterine and Perinatal Environment on the Development of Metabolic Syndrome

**DOI:** 10.1371/journal.pone.0063021

**Published:** 2013-05-17

**Authors:** Patricia M. Vuguin, Kirsten Hartil, Michael Kruse, Harpreet Kaur, Chia-Lei Vivian Lin, Ariana Fiallo, Alan Scott Glenn, Avanee Patel, Lyda Williams, Yoshinori Seki, Ellen B. Katz, Maureen J. Charron

**Affiliations:** 1 Division of Pediatric Endocrinology, Children's Hospital at Montefiore, Bronx, New York, United States of America; 2 Division of Neonatology, Children’s Hospital at Montefiore, Bronx, New York, United States of America; 3 Department of Biochemistry, The Albert Einstein College of Medicine, Bronx, New York, United States of America; 4 Department of Medicine, The Albert Einstein College of Medicine, Bronx, New York, United States of America; 5 Department of Obstetrics and Gynecology, Bronx, New York, United States of America; IGBMC/ICS, France

## Abstract

Genetic and environmental factors, including the in utero environment, contribute to Metabolic Syndrome. Exposure to high fat diet exposure in utero and lactation increases incidence of Metabolic Syndrome in offspring. Using GLUT4 heterozygous (G4+/−) mice, genetically predisposed to Type 2 Diabetes Mellitus, and wild-type littermates we demonstrate genotype specific differences to high fat in utero and lactation. High fat in utero and lactation increased adiposity and impaired insulin and glucose tolerance in both genotypes. High fat wild type offspring had increased serum glucose and PAI-1 levels and decreased adiponectin at 6 wks of age compared to control wild type. High fat G4+/− offspring had increased systolic blood pressure at 13 wks of age compared to all other groups. Potential fetal origins of adult Metabolic Syndrome were investigated. Regardless of genotype, high fat in utero decreased fetal weight and crown rump length at embryonic day 18.5 compared to control. Hepatic expression of genes involved in glycolysis, gluconeogenesis, oxidative stress and inflammation were increased with high fat in utero. Fetal serum glucose levels were decreased in high fat G4+/− compared to high fat wild type fetuses. High fat G4+/−, but not high fat wild type fetuses, had increased levels of serum cytokines (IFN-γ, MCP-1, RANTES and M-CSF) compared to control. This data demonstrates that high fat during pregnancy and lactation increases Metabolic Syndrome male offspring and that heterozygous deletion of GLUT4 augments susceptibility to increased systolic blood pressure. Fetal adaptations to high fat in utero that may predispose to Metabolic Syndrome in adulthood include changes in fetal hepatic gene expression and alterations in circulating cytokines. These results suggest that the interaction between in utero-perinatal environment and genotype plays a critical role in the developmental origin of health and disease.

## Introduction

Obesity is a major risk factor for developing Type 2 Diabetes Mellitus (T2DM). Genetic and environmental factors contribute to both. Genome Wide Association Studies (GWAS) have identified several loci associated with body mass index (BMI) [1] and type 2 diabetes (T2DM) [2], however they do not explain entirely the heritability of these disorders [2]. In addition to genetic predisposition and postnatal environment in determining susceptibility to T2DM and Metabolic Syndrome (MetS), exposures in utero (IU) also play a role. Longitudinal studies in monozygotic and dizygotic twins support the idea that environmental factors are critical to the development of obesity [3] and altered glucose homeostasis [4]. Studies in Pima Indians support the concept of a non-genetic transgenerational transmission of T2DM [5]. Siblings born to diabetic mothers have an increased incidence of diabetes than siblings born when the mother was not diabetic [6]. Animal models [7], [8] and epidemiological studies support the Thrifty Phenotype Hypothesis [9] and the Developmental Origins of Health and Disease (DOHaD) [10] which propose that malnutrition during fetal and early life predispose offspring to metabolic disease.

Western style diet, characterized by high intakes of red and processed meat, sugary desserts and drinks, high-fat foods, and refined grains [11], in pregnancy has been associated with decreased birth weight [12], [13]. In animal models, intrauterine exposure to diets high in saturated fat results in features of MetS in offspring [14]. In a non-human primate (NHP) model, maternal high fat diet (HF) resulted in an inflammatory-oxidative stress response and lipotoxicity in fetal liver [15]. We reported previously in a mouse model, that HF IU and lactation (L) decreased birth weight compared to controls [16]. When decreased birth weight was accompanied by catch-up growth, features of MetS developed including increased adiposity and glucose levels [16]. Whether the IU environment interacts with genetic predisposition to augment risk of developing MetS is less well studied.

G4+/− mice have a heterozygous deletion of the insulin sensitive glucose transporter-4 (GLUT4) gene. In male mice, decreased GLUT4 expression is accompanied by increased serum glucose and insulin, reduced muscle glucose uptake, hypertension, and diabetic histopathologies in the heart and liver [17]. Skeletal muscle expression of GLUT4 is low in the fetal period and a continuous induction of GLUT4 mRNA and protein takes place during perinatal development. Similarly, GLUT4 expression in the heart is induced late in fetal life and increases progressively after birth [18], [19].

G4+/− offspring provide a novel genetic “at risk” model to study the effects of an altered IU/L environment on the DOHaD. Additionally, wild-type (WT) littermates provide optimal controls. We hypothesized that increased MetS in offspring exposed to HF IU/L is due in part to fetal adaptations that result in increased hepatic oxidative stress. In addition, we predicted that susceptibility to MetS will be exacerbated in the G4+/− “at risk” offspring compared to WT littermates exposed to HF IU/L.

## Materials and Methods

### Animals and Experimental Design

Animals were housed in a barrier facility and maintained on a 14–10 hr light-dark cycle with *ad libitum* access to chow and water. Twelve to 14 wk old WT female mice (CD1 background) were maintained on a breeding control (C) PicoLab® Mouse Diet #5058 (chemical composition: 9% fat, 20% protein, 53% carbohydrate, 3.59 kcal/g), or switched to HF Bio-Serv Product #F3282 (chemical composition: 35.5% fat as lard, 20% protein, 36.3% carbohydrate, 5.29 kcal/g) 2 weeks prior to mating, throughout pregnancy and lactation (IU/L) as previously described [16]. Females were bred to non-littermate G4+/− males (11 generations backcrossed onto CD1). Pregnancy was confirmed by detection of a copulatory plug and defined as embryonic day (e) 0.5. Animal protocols were approved by the Institute for Animal Care and Use Committee at the Albert Einstein College of Medicine.

### Study 1– Male Offspring

Pups were genotyped shortly after birth [20]. Male pups were weaned onto low fat (LF) control rodent chow: Pico Lab #5053 (C: 4.5% fat, 20% protein, 54.8% carbohydrate, 3.59 kcal/g) at 3 wks of age. The 5053 chow is a lower fat chow compared to the 5058 breeding chow as well as the HF diet used during the IU/L period. Body weights (BW) were measured weekly. WT and G4+/− male offspring were studied between 6–13 wks of age.

### Study 2– Fetal

Pregnant mice (WT C, n = 16; WT HF, n = 21) were sacrificed on e18.5. Litter number, placental and fetal weights and crown-rump length (CRL) were recorded as well as the number of abnormal or dead pups per litter. Fetuses (C diet, n = 228; HF diet, n = 280) were euthanized by decapitation immediately following dissection from the uterine horn. Fetal serum was collected in heparinized capillary tubes. Fetal livers were dissected and frozen in liquid nitrogen for later analysis. Genotyping and sex determination of fetuses were performed as previously described [16], [21]. No differences were found when the data was analyzed based on fetal sex with regards to weight, serum markers, hepatic glycogen, triglycerides and cholesterol determination, and hepatic gene expression (data not shown).

### Serum Analysis

For fetal serum metabolite and cytokine analysis, samples were pooled by fetal genotype, sex and IU diet. Each sample was pooled from 4–10 fetuses from 4–9 litters/diet. Commercially available kits were used for measuring serum insulin, non-esterified fatty acids (NEFAs) (Wako Chemicals, Neuss, Germany), adiponectin (Linco Research, St. Charles, MO), triglycerides (TG) [16], PAI-1 (Lincoplex, determined by the DRTC core at Einstein), and β-hydroxybutyrate (β-HB) levels (Stanbio Laboratory, Boerne, TX).

Cytokines were measured using the MILLIPLEX MAP Mouse Cytokine/Chemokine - Premixed 32 Plex (Millipore Corporation, Billerica, MA). The cytokine kit analyzed: eotaxin, granulocyte colony-stimulating factor (G-CSF), granulocyte-macrophage colony-stimulating factor (GM-CSF), interferon γ (IFN), interferon γ-induced protein 10 kDa (IP-10), interleukins: IL-1α, IL-1β, IL-2, IL-4, IL-5, IL-6, IL-7, IL-9, IL-10, IL-12 (p40), IL-12 (p70), IL-13, IL-15, IL-17, keratinocyte chemoattractant (KC), leukocyte inhibitory factor (LIF), lipopolysaccharide induced CXC chemokine (LIX), macrophage colony stimulating factor (M-CSF), monocyte chemotactic protein-1 (MCP-1), macrophage inflammatory proteins (MIP-1α, MIP-1β, MIP-2), monokine induced by gamma-interferon (MIG), regulated upon activation normal T-cell expressed, and presumably secreted (RANTES or CCL5), tumor necrosis factor α (TNF-α), and vascular endothelial growth factor (VEGF). The concentration range for the standard curve was between 3.2 pg/mL (lower limit of detection) and 10,000 pg/mL (upper limit of detection). The inter-assay and intra-assay coefficients were 4.2 - 21.2 and 3.0 - 22.6%, respectively.

### Glycogen Determination

Glycogen was measured as previously described [22]. Briefly, fetal liver (n = 6–8 livers/diet/genotype) was digested in 0.05M NaOH at 95°C for 30 min. Glycogen was precipitated with 1.1 volume 95% ethanol. The glycogen precipitates were dissolved in water and analyzed by the phenolsulfuric acid colorimetric method [22].

### Triglyceride (TG) and Cholesterol Determination

Liver TG content (n = 6–8 livers/diet/genotype from 6–8 litters) was determined using a method previously described [23]. Final TG concentration was determined using the TG reagent from Roche (Basel, Switzerland; triglyceride/GB 450032). Liver total cholesterol was measured using reagent no. 704036 from Roche Diagnostics (Indianapolis, IN).

### Intraperitoneal Insulin and Glucose Tolerance Tests

For insulin tolerance tests (ITT), 6 hr fasted mice received an intraperitoneal (i.p.) injection of insulin (0.75 U/kg BW) (Humalog, Lilly, Indianapolis, IN) (n = 5/group randomly selected from four-five litters per group, males: 7–8 wk). For glucose tolerance tests (GTT), overnight fasted mice received an i.p. injection of D-(+)-glucose (1.5 g/kg BW) (n = 5/group randomly selected from four-five litters per group, males: 7–8 wk). Blood glucose levels were measured using a glucometer (Precision Q.I.D., a gift from Abbott Laboratories, Chicago, IL) at the indicated times [16].

### Body Composition

Percent body fat (n = 5/group randomly selected from four-five litters per group, males: 7–9 wk) was determined either using MRI as previously described [16] or using an ECHO magnetic resonance spectroscopy instrument (Echo Medical Systems, Houston, TX).

### Blood Pressure Measurements

Systolic and diastolic blood pressures (SBP and DBP) in mmHg were measured using an indirect tail-cuff volume pressure recording sensor method. This method incorporates a specially designed differential pressure transducer that measures the SBP and DBP by determining the blood volume in the tail. Up to 6 mice were placed in individual tube restrainers on a temperature-controlled platform. Tails remained exposed for positioning of volume-pressure sensor and tail-cuff inflation/deflation. Blood pressure was recorded every 20 seconds using a PC-integrated CODA6 system (Kent Scientific Corp). Mice were trained at least twice before measurements were acquired. Each measurement session consisted of 15–17 cycles (one cycle = one BP reading) and the 5–8 acclimation cycles which assist in animal adaptation.

### Quantitative Real Time-PCR Analysis

mRNA extraction and cDNA preparation was performed using fetal liver (n = 12–15 diet/genotype) as previously described [16], [24], [25]. Quantitative real-time PCR (qRT-PCR) was the method of choice to determine the expression of genes of interest [26]. The ratio of relative expression of the target gene in fetal liver based on genotype and diet was then calculated as 2{Δ}{Δ}Ct, where {Δ}{Δ}CtΔ{Δ}Ct X fetal liver Δ {Δ}Ct control fetal liver. Each sample was measured in triplicate to assess technical variability [26].

#### Selection of genes of interest

The selection of genes was based on a literature search for genes associated with an altered IU mileu [15], [27]. Four commonly used housekeeping genes, ubiquitin, ß-actin (ACTB), hypoxanthine guanine ribotransferase (HPRT1), and 36B4 [28] were used for normalization based on their stable characteristics, as previously described [26]. Furthermore, to prevent false positive results, none of the housekeeping genes used werefrom the same pathway.

### Data Analysis

Data represents the mean ± SEM. Statistical analyses were performed using JMP IN 5.1 software (SAS Institute, Cary, NC) or GraphPad Prism software version 5.00 for Windows (GraphPad Software, San Diego California USA, www.graphpad.com). ANOVA was used to test the difference between the means of two (*t-test)* or more groups. For the cytokine analysis, ANOVA as well as linear regression models were used to evaluate cytokine levels in relation to fetal genotype, sex, body weight at e18.5, and IU diet using separate regression models for each individual metabolite/cytokine using the JMP 7.0 statistics package (SAS).

## Results

### Study 1

#### HF IU/L altered postnatal growth and increased adiposity

**Figure 1:**
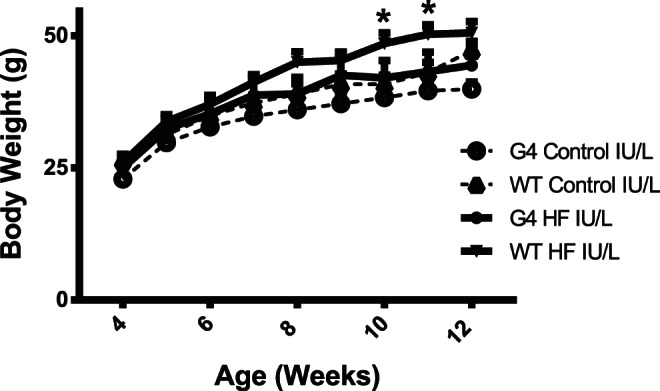
Body weight (BW) gain for WT and G4+/− male offspring exposed to Control or HF IU/L. BW, measured weekly after birth, depicted in the figure from week 4 to 12 (n = 5–12/group). Data represents mean ± SEM; *p<0.02 WT C IU/L vs. WT HF IU/L.

We previously reported that mice exposed to HF IU had decreased weight at birth compared to mice exposed to C IU/L [16]. In addition, HF IU was associated with decreased numbers of pups/litter at birth when compared to C diet [16]. Although there was a tendency for HF IU offspring to weigh less, there was no significant difference in weights at weaning (14.0±0.9, 11.6±0.4, 13.5±1.1 and 11.6±0.5 mg/dl for WT C IU/L, WT HF IU/L, G4+/− C IU/L, and G4+/− HF IU/L respectively, p = NS). At 10 wks of age WT mice exposed to HF IU/L and weaned to LF chow weighed significantly more than WT C IU/L (40.8±2.4 vs. 48.5±2.0, WT C IU/L vs. WT HF IU/L respectively, p = 0.02) (Figure 1). In contrast, at 10 wks of age G4+/− HF IU/L offspring had a similar BW as G4+/− C IU/L (38.2±1.4 and 42.0±3.2 g, G4+/− C IU/L and G4+/− HF IU/L respectively, p = NS) (Figure 1). From 8wks of age on, WT HF IU/L had a tendency to weigh more than G4+/− HF IU/L although this did not reach statistical significance.

**Figure 2:**
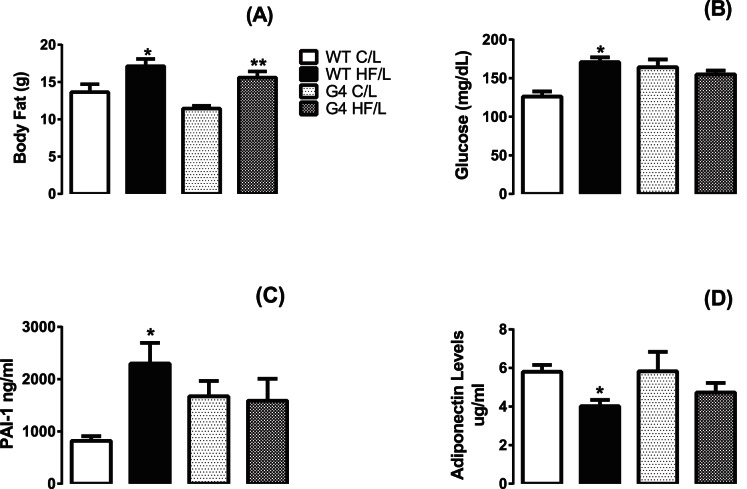
Increased body fat, glucose and PAI-1 and decreased adiponectin levels in 6 wk old WT offspring exposed to HF IU/L. Body fat was determined by MRI (A). Data represent mean ± SEM, n = 5 per group, *p<0.05 vs. C IU/L, **p = 0.027. Fed state blood was collected via retro-orbital sinus between 11PM and 1AM. Plasma glucose (B), PAI-1 (C), and adiponectin (D) levels were determined as described in the Methods section. Data represent mean ± SEM, n = 9–14 per group, *p<0.05 vs. WT C IU/L.

Compared to C IU/L both WT and G4+/− HF IU/L offspring had increased adiposity at 7–8wk of age (Figure 2A). Fat mass was increased 25% in WT HF IU/L and 36% in G4+/− HF IU/L offspring compared to WT C and G4+/− C IU offspring respectively. Despite similar adiposity at 7–8 wks of age, there was a small difference in BW between HF IU/L WT and G4+/− at 11 weeks of age (50.2±0.5, and 43.2±4 g for WT HF IU/L and G4+/− HF IU/L, respectively, n = 5–11/genotype/diet; p = 0.06) that could be explained by the accelerated BW gain in HF IU/L WT (Figure 1). By 12 wks of age, differences in BW between the G4+/− and WT HF IU/L were no longer significant.

#### HF IU/L increased glucose and PAI-1 levels and decreased adiponectin levels in serum of 6 wk old offspring

Fed glucose (Figure 2B) and PAI-1 levels (Figure 2C) were increased and adiponectin (Figure 2D) levels were decreased in WT HF IU/L offspring at 6 wks of age. Insulin, TG, glycerol and NEFA levels were not significantly different between groups at this age (Table 1). HF IU/L did not alter the serum profile of G4+/− offspring.

**Table 1:** Metabolic phenotype of 6 week old male offspring.

	WT C IU/L	WT HF IU/L	G4+/− CIU/L	G4+/− HFIU/L
Insulin (ng/ml)	7.3±1.5	5.5±0.7	12.5±3.2	6.9±1.1
Triglyceride (mg/dl)	102.8±11.6	106.3±10.9	109.3±9.0	101.6±14.3
Glycerol (mg/dl)	82.4±6.8	65.9±6.1	69.8±4.6	66.2±9.6
NEFA(µEq/ml)	2.9±0.09	3.3±0.1	3.1±0.1	2.9±0.22

WT and G4+/− offspring exposed to C or HF IU/L diet, n = 8–16/genotype/diet. No differences were seen in insulin, triglycerides, glycerol and NEFA levels among the groups.

#### HF IU/L impaired insulin and glucose tolerance

**Figure 3:**
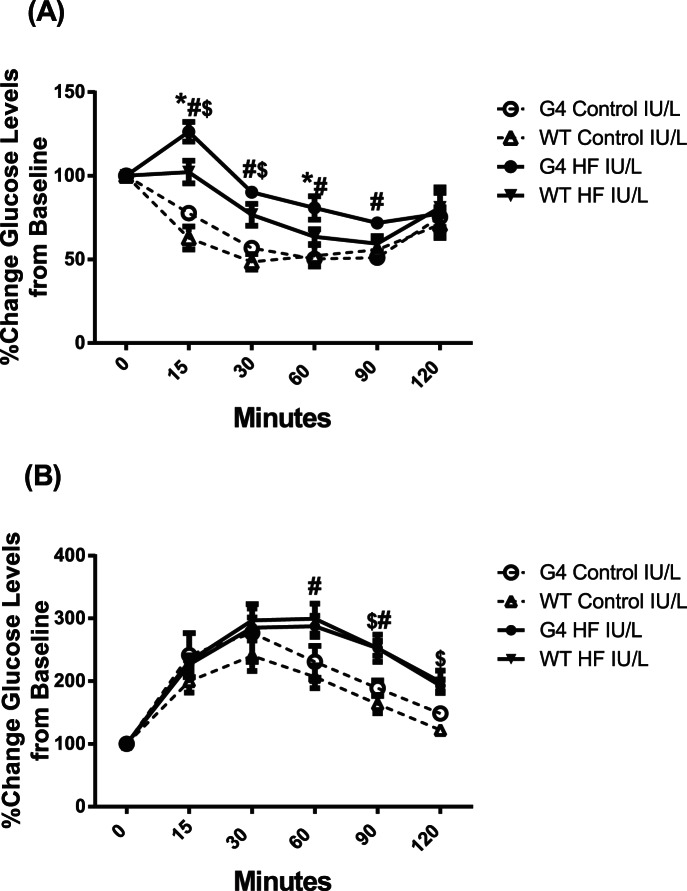
Impaired insulin and glucose tolerance IPGTT in WT and G4+/− mice exposed to HF IU/L. (A) ITT, following a 6 hr fast blood glucose levels were assessed at the indicated times following an i.p. injection of 0.75 U/kg insulin. Values are represented as the percent of t = 0 glucose levels, *p<0.05 G4+/− HF IU/L vs. WT HF IU/L; #p<0.02 G4+/−HF IU/L vs. G4 C IU/L; $p<0.05 WT HF IU/L vs. WT C IU/L. (B) GTT was performed following an overnight fast, mice were i.p. injected with 1.5 g/kg glucose and blood glucose levels determined at the indicated times, n = 5 per group. Values are represented as the percent of t = 0 glucose levels, #p<0.05 G4+/−HF IU/L vs. G4+/− C IU/L; $p<0.05 WT HF IU/L vs. WT C IU/L.

Insulin tolerance tests demonstrated that both WT and G4+/− HF IU/L offspring displayed blunted glucose clearance in response to insulin (Figure 3A). This appeared to be a result of an initial delay in the ability of insulin to stimulate glucose disposal as glucose levels did not decrease at 15 min. Fasting glucose levels were not significantly different between groups (221±10, 192±9, 200±10 and 221±10 mg/dl for WT C IU/L, WT HF IU/L, G4+/− C IU/L, and G4+/− HF IU/L respectively, p = NS).

Glucose clearance in response to a glucose bolus was blunted in HF IU/L compared to C IU/L offspring (Figure 3B). Initial glucose levels were not different between groups (181±22, 142±6, 171±19, and 161±9 mg/dl for WT C IU/L, WT HF IU/L, G4+/− C IU/L, and G4+/− HF IU/L, respectively, p = NS). Consistent with a hemizygous mutation in GLUT4, G4+/− C IU/L offspring displayed minor alterations in glucose tolerance compared to WT as the area under the glucose curve was higher for G4+/− C IU/L compared to WT C/L offspring (912.8, 1230, 1062 and 1198 mg. 1^−1 ^hr^−1^ for WT C IU/L, WT HF IU/L, G4+/− C IU/L, and G4+/− HF IU/L respectively, p = NS).

#### HF IU/L elevates blood pressure in G4+/− offspring

At 13 wks of age G4+/− HF IU/L offspring displayed a statistically significant increase in SBP compared to all other groups (92±2, 94±1, 94±2, and 99±7 mmHg for WT C IU/L, WT HF IU/L, G4+/− C IU/L, and G4+/− HF IU/L, respectively, n = 13–15/genotype/diet; p = 0.01; G4+/− HF IU/L vs. other genotypes/diets). In contrast, DBP was not statistically significantly different between groups (69±2, 65±2, 70±2 and 69±2 mmHg for WT C IU/L, WT HF IU/L, G4+/− C IU/L, and G4+/− HF IU/L, respectively, n = 13–15/genotype/diet; p = 0.09).

To determine whether increased susceptibility to features of MetS in offspring exposed to HF IU/L had its etiological origins during fetal development, a second study was performed to determine fetal responses to HF IU.

### Study 2

#### HF IU altered fetal body weight but not litter number

As previously published maternal body weight and visceral fat pad weights did not differ between the two diets [16]. No difference in number of fetuses per litter was seen (14±2.6, 13±1.4, WT C, WT HF, n = 56–74 fetuses from 5–10 litters per group). HF IU decreased fetal weight, crown rump length (CRL) and placenta weight (Table 2). Neither fetal sex nor genotype affected BW, CRL or placental weight (data not shown).

**Table 2:** Metabolic phenotype of e18.5 fetuses.

	WT C IU	WT HF IU	G4+/− C IU	G4+/− HF IU
BW (g)	1.38±0.03	1.24±0.18*	1.35±0.04	1.21±0.04^#^
Placenta (g)	0.105±0.004	0.111±0.004	0.114±0.003	0.097±0.004^#$^
CRL (cm)	2.52±0.03	2.43±0.02*	2.57±0.03	2.47±0.03^#^
Glucose (mg/dl)	58±3	64±4	54±3	47±6^$^
Insulin (ng/ml)	1.2±0.3	1.4±0.3	1.7±0.3	1.5±0.2
Free Glycerol(mg/dl)	59±11	76±10	59±20	65±9
Total Glycerol(mg/dl)	98±7	105±5	85±10	112±19
TG (mg/dl)	39±6	32±7	27±10	47±13
β-HB (mM)	0.65±0.12	0.57±0.08	0.38±0.15	0.43±0.09
IFNγ (pg/ml)	7.8±1.4	7.2±1.2	3.6±1.0	7.0±0.8^#^
M-CSF (pg/ml)	33.9±12.6	38.7±10.9	33.0±9.5	72.5±7.6^#$*^
Rantes (pg/ml)	34.0±1.6	27±1.4	21.5±1.2	30.7±1.0^#^
MCP-1 (pg/ml)	240±104	257±90	184±78	418±63^#^
Hepatic TG	5.35±1.51	4.38±0.36	4.19±0.74	4.14±0.90
Cholesterol	0.44±0.04	0.75±0.30	0.33±0.07	0.41±0.18
Glycogen	2.98±0.22	2.52±0.72	1.95±0.40	1.31±0.11

WT and G4+/− fetuses at embryonic day 18.5 exposed to C or HF IU diet, n = 5–12/genotype/diet; *p<0.05 vs. WT C; ^#^ p<0.05 vs. G4+/− C;^ $^ p<0.05 vs. WT HF IU. Hepatic triglyceride, cholesterol and glycogen content in µg/mg tissue.

#### Serum glucose and cytokine levels are dependent on fetal genotype and IU diet

Glucose levels were significantly increased by 35% in WT HF IU fetuses compared to G4+/− HF IU. Serum insulin, β-HB, TG and glycerol levels were not different between groups (Table 2).

Since hepatic inflammation and lipotoxicity accompanied exposure to HF IU in a NHP model [15], we evaluated inflammation by measuring serum levels of 32 cytokines (Table 2). A total of 16 cytokines were detected in all samples. Cytokines not detected by the assay were: GM-CSF; IL1β, 2–5, 7, 9, 10 and 12; VEGF; MIP-2; MIP-1α and LIF.

Increased levels of IFNγ, M-CSF, RANTES, and MCP-1 were measured in G4+/− HF IU compared to G4+/− C IU (Table 2). Following adjustment for fetal genotype, fetal sex, body weight and diet; fetal genotype was associated with increased concentrations of TNFα in G4+/− compared to WT (3.8±0.3 vs. 4.6±0.3 pg/ml, WT vs. G4+/−, p = 0.016) and IP-10 in WT compared to G4+/− fetuses (234±17 vs. 205±17 pg/ml, WT vs. G4+/−, p = 0.04). Body weight was inversely associated with M-CSF (p = 0.002), MCP-1 (p = 0.008), and RANTES levels (p = 0.001), suggesting that the smallest fetuses have the high plasma levels of inflammatory markers.

#### Hepatic glycogen, TG and cholesterol levels are not altered by fetal genotype or IU diet

Despite differences in serum glucose levels, no difference in hepatic glycogen content was observed. Additionally, no difference in hepatic TG or cholesterol was measured (Table 2).

#### Gene expression in HF fetal liver is consistent with decreased insulin action and increased gluconeogenesis

Dysregulated hepatic insulin signaling, gluconeogenesis, inflammation and oxidative stress have all been implicated in the pathogenesis of MetS. To determine whether HF altered these processes in fetal liver we quantified expression of genes involved in these pathways. Sequence-specific primer pairs used are provided in Table 3.

**Table 3:** Relative Hepatic mRNA Expression in WT and G4+/− fetuses exposed to HF IU Compared to C IU.

Gene Name	Gene Symbol	Gene Sequence(Forward and Reverse)	WT HF vs. WT C	G4+/− HF vs. G4+/− C
**A. Glucose metabolism**				
Phosphoenolpyruvate carboxykinase 1	PCK1	TGCAGCCTACAATCTGCTCCGTCAAGTGTGCGTAGTTCTGA	3.5***	5.9***
Phosphofructokinase 1	PFK	TGCAGCCTACAATCTGCTCCGTCAAGTGTGCGTAGTTCTGA	1.5*	2**
Glucose-6-phosphatase, catalytic subunit	G6PC	GCCTCCTGTCGGATACAGAATGCACCGCAAGAGCATT	2.3**	3.7***
Glycogen synthase kinase 3 alpha	GSK3	TCAAGCCCCAGAATTTGCTCCCCGAACCAGCTGCTTT	2*	1.4*
Forkhead box A2	FOXA2	TAGCGGAGGCAAGAAGACCCTTAGGCCACCTCGCTTG	1.3*	1.4*
Sirtuin 1	SIRT1	GCCAAACTTTGTTGTAACCCTGTATGGTGGCAACTCTGATAAATGAA	1.4*	NS
**B. Insulin action**				
Insulin receptor substrate 1	IRS1	CGAGAGCTGTTTCAACATCAACACGCGGCAATGGCAAA	NS	NS
Insulin receptor substrate 2	IRS2	CAGAGCAAGAACCTGACTGGTGTATGGCTGTTCGCAATTGAGCTT	NS	NS
Solute carrier family 2 facilitated glucose transporter member 2	SLC2A2	GTGTGCAGCAGCCTGTGTTTGACTGGAGCCCTCTTGATG	NS	12***
Insulin like growth factor binding protein -1	IGFBP-1	TCCTGTGGAACGCCATCAGTTCTTGAGGTCGGCGATCTC	1.6*	2.6*
**C. Oxidative stress and inflammation**				
Suppressor of cytokine signaling 3	SOCS3	CCACCCTCCAGCATCTTTGTTCCAGGAACTCCCGAAT	1.8**	1.3*
Tumor necrosis factor α	TNFα	GGCACTCCCCCAAAAGATGAGGAATGAGAAGAGGCTGAGACA	1.8*	1.3*
Serpin peptidase inhibitor, clade E, member 1	SERPINE1	TTGTCCAGCGGGACCTAGAGAAGTCCACCTGTTTCACCATAGTCT	4.1**	2.8*
Thioredoxin	TRX	CAGCCTCTGGCACATTTCCTGTTCGGCTTCTGGTTTCCTTT	−0.53*	−0.5
V-maf musculoaponeurotic fibrosarcoma oncogene homolog F	MAFF	GGTGGAAGAGGCTTTGGATTGGTCTCACAAGGCCACACCTAGTC	−0.40*	−0.48*
Dual specificityphosphatase 1	DUSP	GTGCCTGACAGTGCAGAATCCACTGCCCAGGTACAGGAAG	−0.37*	−16
Intercellular adhesion Molecule 1	ICAM	CCCCGCAGGTCCAATTCCAGAGCGGCAGAGCAAAAG	NS	2.9*
Heme oxygenase (decycling) 1	HMOX1	CTGCTAGCCTGGTGCAAGACCAACAGGAAGCTGAGAGTGA	4.26**	NS
Retinol dehydrogenase 12	RDH12	CCCACTTTGGAGTCAACCACGCTATTGAGGAAAGGTTGACCA	NS	−0.4**
**D. Cortisol metabolism**				
Nuclear receptor subfamily 3, group C, member 1	NR3C1	TGGAGCTACAGTCAAGGTTTCTGCTTGGAATCTGCCTGAGA	NS	NS
Hydroxysteroid (11-beta) dehydrogenase 1	HSD11B1	GGGAAATGACCCAGCCTATGCGTGGAAAAGAACCCATCCA	NS	NS
Hydroxysteroid (11-beta) dehydrogenase 2	HSD11B2	CGGGCAGTTCCTGAATTCACGCATCGATGATGGCATCTACA	NS	NS
**E. Lipid metabolism**				
Fat-specific protein-27	CIDEC	CGCATCGTGAAGGAGATCGTAGGTGCCAAGCAGCATGTG	1.89*	NS
3- hydroxyl-3-methylglutaryl- CoA synthase 2 (mitochondrial)	HMGCS2	GCGGTCTCCTTGCTTTGCTCACCGGTTCCTCCTTCAG	NS	NS
3- hydroxyl-3-methylglutaryl- CoA reductase	HMGCR	CACTGACATCGAGGGCATAGATACCATAGCGACCATCCAGTAGCT	NS	NS
Sterol regulatory element binding transcription factor 2	SREBF2	CACCAGCTGCACATCACAGACTCGGCCAGGTTCACAG	NS	−0.3**
Sterol-C5-desaturase	SC5D	GTCCTCGCCCTTATCTGATGGTCCTCGCCCTTATCTGATG	NS	NS
Cytochrome P450,family 3, subfamily A	CYP3A	TTTCAGCTCTCTCACTGGATACAT CCAGGAATCCCCTGTTTCTTA	NS	NS
Fatty acid synthase	FASN	GCCTCCTGTCGGATACAGAACTCACGGAGTTCTGCCAGTTC	NS	NS

Gene expression was determined for genes involved in: (A) glucose metabolism, (B) insulin action, (C) oxidative stress and inflammation, (D) cortisol and (E) lipid metabolism. Fold change indicates the increase in mRNA measured by qRT-PCR in HF IU compared to C IU in WT and G4+/− fetal liver (n = 5–11/group) at e18.5 *p<0.05, **p<0.01, ***p<0.001 in HF IU vs. C IU diet; NS  = non significant.

Expression of the proximal insulin receptor signaling genes 1 and 2 (IRS-1 and 2), were not affected by genotype or diet (Table 3). The solute carrier family 2 facilitated glucose transporter member 2 (Slc2a2), the bi-directional high capacity and low affinity glucose transporter, mRNA expression was increased 12-fold in fetal liver of G4+/− HF IU compared to G4+/− C IU. No difference in Slc2a2 expression was seen in WT offspring (Table 3). Gene expression of forkhead transcription factor A2 (FOXA2), glycogen synthase kinase-3 alpha (GSK3α), and insulin like growth factor binding protein-1 (IGFBP-1), proteins whose activities are all regulated by insulin, were increased with HF IU compared to C IU liver (Table 3).

HF IU, independent of fetal genotype, increased expression of genes involved in gluconeogenesis: phosphoenolpyruvate carboxykinase (PCK1); glucose-6-phosphatase (G6PC) and phosphofructokinase 1 (PFK1) compared to C IU liver (Table 3). Gene expression of the NAD^+^-dependent protein deacetylase (SIRT1), a PGC1α activator that regulates expression of gluconeogenic genes was increased in WT, but not G4+/−, HF IU fetal liver compared with C IU (Table 3).

#### Fetal genotype and IU diet alter expression of genes involved in lipid metabolism

Since dyslipidemia is a feature of MetS, we determined expression of genes involved in cholesterol and fatty acid synthesis. WT HF IU fetuses exhibited significantly increased mRNA expression of murine fat-specific protein-27 (CIDEC) compared to WT C IU. G4+/− HF IU fetal liver had significantly decreased expression of sterol regulatory element binding transcription factor 2 (SREBF2) compared with G4+/− C IU (Table 3). Neither HF IU nor fetal genotype altered expression of 3- hydroxyl-3-methylglutaryl- CoA synthase 2 (mitochondrial) (HMGS), 3- hydroxyl-3-methylglutaryl- CoA reductase (HMGR), sterol-C5-desaturase (SCD5), Cytochrome P450, family 3, subfamily A (CYP3A) and fatty acid synthase (FAS) (Table 3).

#### Gene expression in HF fetal liver is consistent with inflammation and oxidative stress

Independent of genotype, HF IU increased hepatic mRNA levels of several inflammatory cytokines: cytokine signaling protein 3 (SOCS3); tumor necrosis factor α TNFα; and serpin peptidase inhibitor, clade E, member 1 (Serpine 1) (Table 3). In addition, HF IU decreased expression of genes involved in the cytoprotective antioxidant response: thioredoxin (TXN); musculoaponeurotic fibrosarcoma oncogene homolog F (v-maf); and dual specificity protein phosphatase 1 (DUSP 1) (Table 3).

In G4+/− HF IU liver, mRNA expression of the pro-inflammatory intercellular adhesion molecule (ICAM) was increased while expression of retinol dehydrogenase 12 (RDH12) was decreased compared to G4+/− C (Table 3). In WT HF liver, mRNA expression of heme oxygenase 1 (HMOX1) was increased compared to WT C (Table 3).

Neither HF IU nor fetal genotype altered expression of nuclear receptor subfamily 3, group C, member 1 (glucocorticoid receptor, NR3C1) or the critical enzymes that catalyze cortisol metabolism (hydroxysteroid- 11-beta dehydrogenase 1-HSD11B1 and hydroxysteroid- 11-beta dehydrogenase 2-HSD11B2) (Table 3) suggesting that corticosteroid excess is not the mechanism associated with DOHaD in our model [29].

## Discussion

Alterations in the IU and perinatal environment have a significant impact on fetal development and susceptibility to MetS in adult life. Given the genetic contribution associated with T2DM and obesity we sought to determine the effect of maternal HF feeding on a mouse model genetically predisposed to develop features of MetS, the G4+/− mouse [1], [2], [17]. As previously published, HF feeding for two weeks prior to mating and during pregnancy did not result in maternal obesity [16]. This model therefore allows us to investigate the interaction between HF exposure IU/L and genotype in the absence of any confounding effect of maternal obesity.

WT and G4+/− mice exposed to HF IU were smaller at e18.5 (decreased fetal weight and CRL) and developed features of MetS compared to mice exposed to C IU/L including decreased glucose and insulin tolerance and increased adiposity. Interestingly, MetS developed despite animals being weaned onto a low fat diet, suggesting that “permanent adaptations" occur in the IU environment that may determine future physiological features, regardless of good dietary and exercise habits. Future studies will address the impact these adaptations may have on susceptibility to MetS when mice are weaned onto a high fat diet.

Reduced fetal/birth weight in response to HF IU has been reported in some studies [15], [30], [31] but not others [27], [32], [33], [34]. Some reasons for these discrepancies include: duration of HF exposure; presence/absence of maternal obesity; diet composition; and species studied. Low birth weight or normal birth weight accompanied by rapid weight gain during the first year of life both increase the risk of obesity [35]. In addition, small size at birth correlates with increased fat mass [36]. Increased postnatal adiposity plays a role in the pathogenesis of the MetS [37]. In this study HF exposure during pregnancy and lactation increased offspring adiposity independent of genotype. Because HF exposure occurred during both pregnancy and lactation we cannot conclude whether there is a critical period of exposure to HF that determines the trajectory of postnatal growth.

Although both G4+/− and WT HF IU/L offspring developed features of MetS, genotype dependent differences were observed. In WT offspring, HF IU/L increased fed serum glucose and PAI-1 levels and decreased adiponectin levels consistent with insulin resistance, inflammation and obesity [38], [39]. G4+/− C IU/L and G4+/− HF IU/L mice had a similar serum profile to WT HF IU/L offspring. Increased glucose levels in G4+/− C IU/L mice are, most probably, a result of decreased glucose transport into GLUT4 expressing tissues such as skeletal muscle and adipose tissue [17], [40]. This data demonstrates that, as early as 6 wks of age, WT HF IU/L offspring exhibit a metabolic profile similar to G4+/− C IU/L mice, a genetic model of MetS.

HF IU/L did not exacerbate the serum profile in G4+/− mice, but G4+/− HF IU/L offspring did exhibit increased SBP compared to WT littermates. This data indicates that hypertension, a feature of MetS, is exacerbated by exposure to HF IU/L in mice with a hemizygous lesion in GLUT4 (GLUT4+/−).

To begin to address the etiology of MetS in response to HF IU/L the metabolic and molecular effects of HF IU on fetal liver were investigated. Liver was selected as it plays a critical role in regulating metabolic processes in response to nutrient availability and has been demonstrated to be highly susceptible to programming IU [41]. Evidence suggests that alterations in the IU environment programs epigenetic modifications in liver that may impact metabolism. By assessing gene expression, our aim was to identify pathways that maybe targets of epigenetic modifications in utero. Gene expression data suggests that HF IU produces a phenotype in fetal liver similar to that observed with fasting.

Fetal serum ketones, glycerol and TG levels, and hepatic glycogen levels were not altered in response to HF IU suggesting that alterations in gene expression were a compensatory adaptation. Decreased fetal serum glucose levels in G4+/− HF fetus compared to WT HF may be due to the 12-fold increase in gene expression of Slc2a2, the glucose transporter GLUT2 (p<0.01) in G4+/− HF IU liver. Increased Slc2a2 expression may be a compensatory adaptation by the G4+/− “at risk” liver and may explain why G4+/− HF IU offspring maintained similar glucose levels to G4+/− C IU offspring. Alternatively, GLUT4 expression, which has been detected in mouse placenta as early as e12 [42], may be decreased in G4+/− placenta resulting in decreased fetal-placenta glucose transport.

Expression of genes involved in glycolysis (PFK) and gluconeogenesis (PCK1, G6Pase) were increased in HF fetal liver. Increased PCK1 gene expression increases basal hepatic glucose production (HGP), triggering impaired glucose tolerance [43]. Increased expression of genes involved in gluconeogenesis and glycolysis accompany hepatic insulin resistance in rodent models of IU programming [15], [41], [44].

Although changes in HGP may not be directly inferred from gene expression data [45], [46] changes in fetal hepatic gene expression are consistent with increased HGP and may, in part, explain the increase in fed glucose levels observed in 6 wk old WT HF IU offspring. In a NHP model, chronic maternal consumption of HF increased expression of gluconeogenic genes. This was accompanied with fetal hepatic lipid deposition [15].

In contrast to that NHP model [15], increased lipid (TG and cholesterol) accumulation in HF fetal liver was not observed. Despite no change in hepatic TG levels, expression of CIDEC mRNA was increased in WT, but not G4+/− HF fetuses. CIDEC is associated with the formation of lipid droplets. Proinflammatory genes such as IFN-γ, which was upregulated in G4+/− HF fetal liver, repress CIDEC expression in adipocytes [47]. Expression of the transcription factor SREBF2, which regulates expression of genes involved in cholesterol synthesis [48], which is associated with insulin resistance [49], was decreased in G4+/− HF IU fetal liver compared with G4+/− C IU. Similar to CIDEC, expression of SREBP is also regulated by inflammation and HF [50], [51] suggesting that altered gene expression between WT and G4+/− HF fetuses could be the result of a different inflammatory response to HF IU.

Fetal lipid accumulation is thought to be primarily maternally derived since rates of *de novo* lipogenesis in fetal liver are low [52], [53], [54]. In a C57Bl/6 mouse model of acute maternal HF feeding, more lipids were transported to the fetus when more lipids were consumed by the mother [55]. The amount of lipid consumed directly correlated with fetal growth [55]. In our model HF IU fetuses were smaller than C IU, this combined with the absence of hepatic TG accumulation, suggests that the phenotype observed in our model may be a result of decreased maternal lipid transfer. Consistent with the NHP model, 2 wks HF in increased maternal serum glycerol and NEFA levels suggesting increased lipolysis, however maternal serum TG levels were decreased [16]. Therefore, it is possible that in our CD1 model, maternal HF results in altered availability of lipids that affect the growth of the fetus.

HF IU increased hepatic expression of genes associated with insulin resistance (TNFα, SOCS3, PAI-1) [56] and cellular stress (TXM, MAFF and DUSP) [57], [58], [59]. We speculate that this gene expression profile could predispose HF IU/L offspring to develop hepatic insulin resistance and steatosis in adult life [60].

Inflammatory cytokines are a proposed link between obesity, insulin resistance and metabolic disease [61]. Markers of oxidative stress and inflammation are increased in livers of NHP fetuses [15] and livers of 15wk old mice exposed to HF IU [62]. Fetal inflammation is associated with several neonatal diseases, such as brain damage and chronic lung disease [63], [64]. However, the role of fetal inflammation in DOHaD remains unresolved. Catalano et al., reported that maternal inflammation does not translate into inflammation of the fetal compartment [65]. In contrast, altered cytokines and inflammatory markers in cord blood have been associated with altered fetal growth associated with placental insufficiency [66], [67], [68], [69]. G4+/− HF IU had increased levels of IFNγ, MCP-1, RANTES and M-CSF compared to G4+/− C IU. In contrast, WT HF IU serum levels were not significantly different than WT C IU. Elevated chemokine levels, such as MCP-1 which contributes to insulin resistance and hepatic steatosis [70], or RANTES which has been associated with obesity [71] may have long term implications for susceptibility to MetS in later life [72].

Inflammatory and redox responses to HF IU was genotype dependent. WT HF fetuses upregulated expression of HMOX1 mRNA, which inhibits leukocyte migration [73]. G4+/− HF fetuses upregulated mRNA expression of ICAM 1, a molecule associated with the recruitment of inflammatory cells [74]. Expression of the retinol dehydrogenase RDH12, a NADP^+^-dependent oxidoreductase [75], was downregulated in G4+/−, but not WT HF IU liver. Both medium-chain aldehydes and retinoids exert biological activities that can lead to cytotoxic effects [75]. These results demonstrate that fetal genotype and IU environment interact to regulate components of the innate immune system.

One possible explanation for the metabolic differences between genotypes could be the developmental regulation of glucose transporters. GLUT4 mRNA and protein are expressed during fetal life in brown adipose tissue, heart and skeletal muscle [18] and is sensitive to alterations in maternal nutrient intake [76]. Differences in the phenotype between genotypes may be related to the specific expression of glucose transporters that play an important role in the regulation of glucose uptake and metabolism under diverse nutritional environments.

## Conclusion

Our model attempts to define the genetic contribution of GLUT4 and its interaction with HF IU/L exposure on the metabolic phenotype. Effects of HF IU/L on the metabolic phenotype were observed in young offspring and significant alterations in fetal hepatic gene expression, as well as low grade inflammation in G4+/− fetuses were detected. Our data support the hypothesis that the etiology of metabolic disease involves an interaction between genetics and the IU and perinatal environment.

We propose that these responses to maternal HF may be necessary to allow the fetus to survive under adverse developmental conditions but ultimately increase the risk for developing MetS during postnatal life. In our model, HF IU was associated with increased expression of genes involved in glycolysis, gluconeogenesis, oxidative stress and inflammation in fetal liver. In addition, we have demonstrated that nature (fetal genotype) modifies nurture (HF IU/L) by showing that offspring genotype increases susceptibility to certain features of MetS in response HF IU/L. By identifying and characterizing gene-environment interactions we have increased opportunities to effectively target metabolic disease intervention strategies.
